# Bacteriocyte dynamics during development of a holometabolous insect, the carpenter ant *Camponotus floridanus*

**DOI:** 10.1186/1471-2180-10-308

**Published:** 2010-12-01

**Authors:** Sascha Stoll, Heike Feldhaar, Martin J Fraunholz, Roy Gross

**Affiliations:** 1 Lehrstuhl für Mikrobiologie, Biozentrum, Universität Würzburg, D-97074 Würzburg, Germany; 2 Lehrstuhl für Verhaltensphysiologie, Universität Osnabrück, D-49076 Osnabrück, Germany; 3 QIAGEN GmbH, QIAGEN Str. 1, D-40724 Hilden, Germany

## Abstract

**Background:**

The carpenter ant *Camponotus floridanus *harbors obligate intracellular mutualistic bacteria (*Blochmannia floridanus*) in specialized cells, the bacteriocytes, intercalated in their midgut tissue. The diffuse distribution of bacteriocytes over the midgut tissue is in contrast to many other insects carrying endosymbionts in specialized tissues which are often connected to the midgut but form a distinct organ, the bacteriome. *C. floridanus *is a holometabolous insect which undergoes a complete metamorphosis. During pupal stages a complete restructuring of the inner organs including the digestive tract takes place. So far, nothing was known about maintenance of endosymbionts during this life stage of a holometabolous insect. It was shown previously that the number of *Blochmannia *increases strongly during metamorphosis. This implicates an important function of *Blochmannia *in this developmental phase during which the animals are metabolically very active but do not have access to external food resources. Previous experiments have shown a nutritional contribution of the bacteria to host metabolism by production of essential amino acids and urease-mediated nitrogen recycling. In adult hosts the symbiosis appears to degenerate with increasing age of the animals.

**Results:**

We investigated the distribution and dynamics of endosymbiotic bacteria and bacteriocytes at different stages during development of the animals from larva to imago by confocal laser scanning microscopy. The number of bacteriocytes in relation to symbiont-free midgut cells varied strongly over different developmental stages. Especially during metamorphosis the relative number of bacteria-filled bacteriocytes increased strongly when the larval midgut epithelium is shed. During this developmental stage the midgut itself became a huge symbiotic organ consisting almost exclusively of cells harboring bacteria. In fact, during this phase some bacteria were also found in midgut cells other than bacteriocytes indicating a cell-invasive capacity of *Blochmannia*. In adult animals the number of bacteriocytes generally decreased.

**Conclusions:**

During the life cycle of the animals the distribution of bacteriocytes and of *Blochmannia *endosymbionts is remarkably dynamic. Our data show how the endosymbiont is retained within the midgut tissue during metamorphosis thereby ensuring the maintenance of the intracellular endosymbiosis despite a massive reorganization of the midgut tissue. The transformation of the entire midgut into a symbiotic organ during pupal stages underscores the important role of *Blochmannia *for its host in particular during metamorphosis.

## Background

Bacteriocyte endosymbiosis is a widespread phenomenon in insects with an estimated 15 to 20% of all insects harboring obligate intracellular endosymbionts [1]. These so-called primary endosymbionts are harbored in specialized cells, the bacteriocytes, as well as in the reproductive tissues to facilitate maternal transmission. Accordingly, they are generally transmitted vertically and show a long history of strict co-evolution with their hosts [2,3]. Bacteriocytes can aggregate and form bacteriomes, organ-like structures in the body cavity of the insect host. Such bacteriomes are frequently associated with the midgut, such as in aphids or tsetse flies, or the fat body as in cockroaches [2,3]. Bacteriocytes can also be found interspersed among cells of host tissues, e.g. within the midgut tissue of carpenter ants, where they are intercalated between midgut cells [4,5]. Within the bacteriocyte the bacteria can either be surrounded by a host derived symbiosomal membrane, e.g. *Buchnera *in aphids [2,6], or they reside in the cytoplasm, e.g. *Blochmannia *in ants [5]. Generally, these bacteria are confined to intracellular locations, although, for instance, *Wigglesworthia*, the primary endosymbiont of tsetse flies, can also be found extracellularly in the milk gland lumen from where the bacteria can infect the developing brood [7]. In contrast to primary endosymbionts, invasion of different tissues is observed frequently for secondary endosymbionts which are not essential for the animals [8]. Early observations indicated that *Blochmannia *may also have a cell invasive capacity, when the bacteria evade from bacteriocytes in the midgut tissue in order to infiltrate the oocytes thus guaranteeing the vertical transmission of the bacteria [9].

Bacteriocyte endosymbionts are frequently observed in animals with a specialized diet lacking nutrients essential for the animals such as aphids or tsetse flies feeding exclusively on plant sap or blood, respectively [10]. There is ample evidence that these mutualists contribute to host nutrition by supplementing the host's diet with, for example, essential amino acids in the *Buchnera*-aphid endosymbiosis or vitamins in the *Wigglesworthia*-tsetse fly interaction. In contrast, ants of the genus *Camponotu*s and related genera such as *Polyrhachis *harboring endosymbiotic *Blochmannia *are generally considered to be omnivorous [11]. However, ants are often limited by nitrogen availability, especially in habitats that are generally poor in nitrogen compounds such as tropical rain forest canopies [12]. *Blochmannia *encodes a functional urease and glutamine synthetase and may therefore be involved in nitrogen recycling. Recently, it was shown that *Blochmannia *upgrades the diet of individual ants by the synthesis of essential amino acids. This is probably also relevant on the colony level by improving the quality of food provided to larvae by care-taking young workers which feed the larvae by trophallaxis [13,14]. Ants are holometabolous animals and these metabolic capabilities of the endosymbiont may be of particular relevance during metamorphosis when the animals are excluded from external food resources. In line with this assumption, massive replication of the bacteria and an upregulation of amino acid biosynthesis genes and urease were observed in particular during pupal stages [14-16].

Very little is known about the cell biology, developmental origin and evolution of bacteriocytes. A general characteristic of such cells appears to be a high degree of polyploidy, possibly reflecting the high metabolic output of these cells [17-20]. The ontogeny of bacteriocytes to date was investigated only in early developmental stages of hemimetabolous aphids, which can reproduce parthenogenetically. The endosymbiotic bacteria are transmitted directly from mother to developing embryos in the blastoderm stage. A two-step recruitment of bacteriocytes was observed in the aphid *Acyrthosiphon pisum *using bacteriocyte specific markers. Bacteriocytes are developed before transmission of maternal bacteria to the aphid embryo and a second population of cells is transformed into bacteriocytes later in development [21]. Nevertheless, in aphid lineages that have secondarily lost the symbiotic bacteria the bacteriocytes were either maintained or their development was initiated but then aborted [21]. The number of *Buchnera *in *A. pisum *may be actively downregulated by the host about two weeks after final ecdysis. The decrease in symbiont number was shown to be correlated with an activation of the lysosomal system of the bacteriocytes [22,23].

Recently, it was shown that in larvae of the holometabolous olive fly *Bactrocera oleae *the vertically inherited endosymbiont *Candidatus *Erwinia dacicola is located intracellularly within midgut cells. After metamorphosis, however, the bacteria have an extracellular location in the foregut. It was consequently suggested that this change in the endosymbiont's location and lifestyle may be related to host metamorphosis [24]. Extracellular endosymbionts residing in the digestive tract of an insect, for example the complex gut microflora of the hemimetabolous termites, are lost with every molting. However, termites much alike ants are social insects and it is thought that behavioral strategies such as trophallaxis or coprophagy allow the vertical transmission of the endosymbiotic community via nutritional exchange between individuals of the termite colony [25].

In previous studies based on light or electron microscopy the distribution of *B. floridanus *containing bacteriocytes during larval and adult stages of its host *C. floridanus *was investigated [4,5,26]. Bacteriocytes were found to have an island-like distribution in the midgut tissue in both life stages examined. So far, the fate of the bacteriocytes and their bacterial inhabitants during pupal stages and the mechanisms of how the symbionts are maintained throughout metamorphosis have not been investigated. At the onset of metamorphosis of holometabolous insects the entire inner larval gut epithelium including the gut content is shed and excreted [27], becoming visible as the meconium (a dark spot at the distal pole of early stage pupae; see below). The epithelial cells are removed by apoptosis and autophagy and their nutrients are reabsorbed by the pupal gut epithelium [27]. Nonetheless, in *C. floridanus *the number of bacteria present in the host constantly increases from larval over pupal stages towards adult workers [15].

Here, we investigated how the symbiosis between the holometabolous ant *C. floridanus *with its primary endosymbiont *B. floridanus *is maintained during metamorphosis. We used fluorescence *in-situ *hybridization (FISH) and direct fluorescence labeling of the bacteria to study the fate of *Blochmannia *and its host cells during larval, pupal and adult life stages of the host.

## Results and Discussion

### Bacteriocyte distribution in larvae of *C. floridanus*

**Figure 1:**
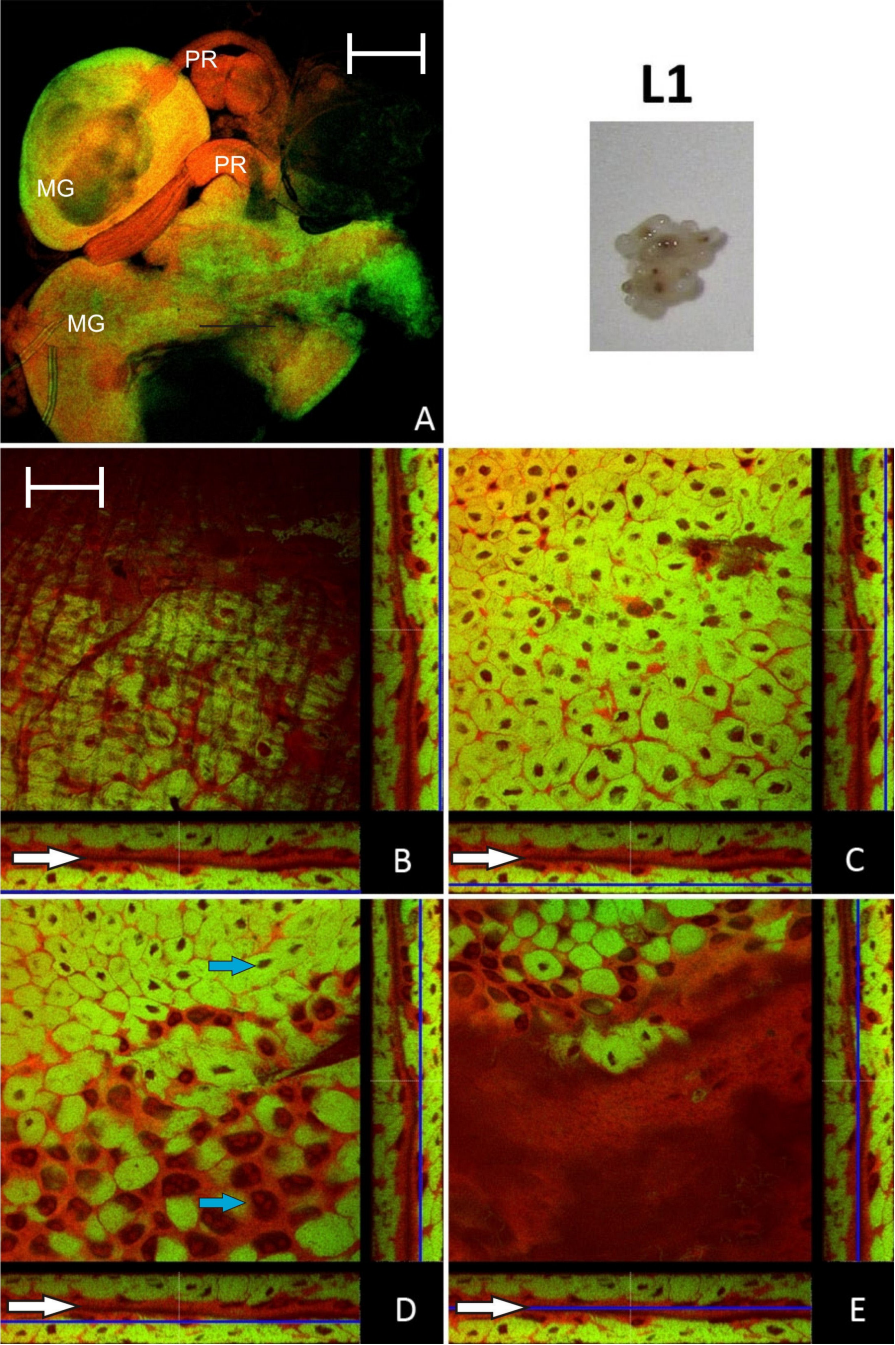
**Larva of stage L1**. A: Overview showing two midguts (MG) and their proventriculi (PR) by confocal laser scanning microscopy. B - E: Four orthogonal views of confocal image stacks of *C floridanus *L1 larva midgut sections. The blue lines in the XZ and YZ stack representations (below and on the right side of each quadratic micrograph) illustrate the position of the image plane (XY). The bacteria-free midgut cells typically have large nuclei and several nucleoli while the bacteriocytes are characterized by small nuclei (blue arrows in D). The bacteriocytes form a nearly contiguous layer surrounding the midgut (B, C) directly underneath of the muscle network (A and Fig. 3). There are no bacteriocytes present in the cell layer lining the midgut lumen (D, E). The midgut lumen is indicated by white arrows. Green label: The *Blochmannia *specific probe Bfl172-FITC; red label: SYTO Orange 83. The scale bars correspond to 220 μM (A) and 35 μM (B - E), respectively.

The distribution of endosymbiont containing bacteriocytes within the host midgut tissue was investigated by FISH and direct fluorescence labeling of bacteria and host cells over different developmental stages. We stained *Blochmannia *with a 16S rRNA specific green-fluorescent oligonucleotide (Bfl172-FITC) and host cells with red-fluorescent SYTO Orange 83 and fluorescence was detected by confocal laser scanning microscopy (CLSM). Figure 1A shows the midgut of L1 larvae at 10 × magnification. Panels B-E show orthogonal views of different optical sections of the image stack of midgut tissue. The Z-positions of the optical midgut sections are indicated by blue lines in the XZ and YZ views below and right of each XY section representation, respectively. The midgut lumen (Figure 1B-E, white arrows) is visible as a continuous space encased by bacteria-free cells. Bacteriocytes can easily be distinguished from other cell types by the densely packed green-fluorescent bacterial mass they contain as well as the relatively small size of their nuclei (Ø 5 - 8 μm) in comparison to the large nucleoli-rich nuclei (Ø 10 - >30 μm) of other midgut cells (Figure 1D; blue arrows). Overall, the analysis of L1 larvae showed that the outer layer of the midgut epithelium comprises largely bacteriocytes, a feature which was also found in a previous *in situ *hybridization study [4]. In contrast, optical sections close to the gut lumen showed an absence of bacteriocytes from the epithelial layer lining the midgut lumen (Figure 1D-E).

**Figure 2:**
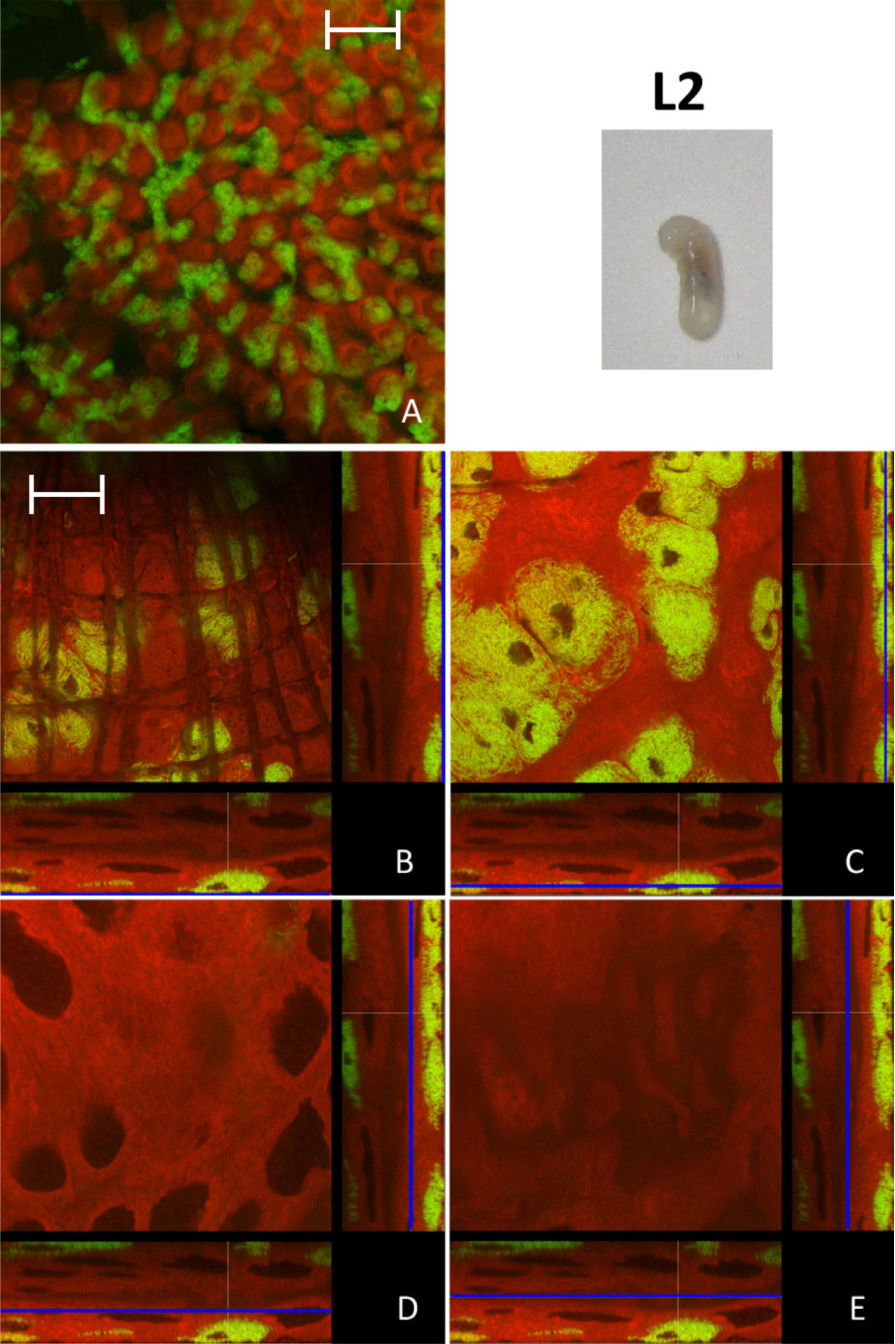
**Larva of stage L2**. Overview (A) and detailed images of different optical sections (B - E) of the midgut of a *C. floridanus *larva (L2) by confocal laser scanning microscopy (for further information regarding the composition of the figure see legend of Fig. 1). The bacteriocytes are located in cell clusters of different size on the outer surface of the midgut (B, C) and the cells lining the midgut lumen are free of bacteria (D, E). Green label: The *Blochmannia *specific probe Bfl172-FITC; red label: SYTO Orange 83. The scale bars correspond to 220 μM (A) and 35 μM (B - E), respectively.

**Figure 3:**
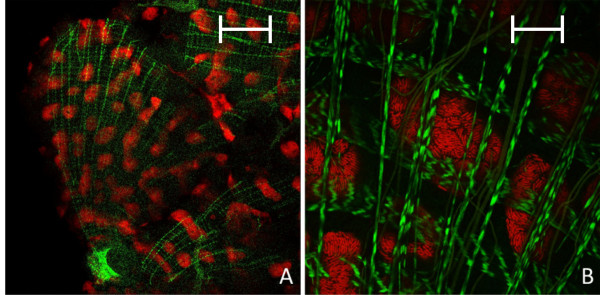
**Overview (A) and detailed image (B) of the actin-stained muscle network surrounding the midgut of a *B. floridanus *larva (L2) by confocal laser scanning microscopy**. Green label: FITC-Phalloidin; red label: The *Blochmannia *specific probe Bfl172-Cy3. The scale bars correspond to 220 μM (A) and 35 μM (B), respectively.

In the last instar larvae (L2) the spatial pattern of bacteriocyte distribution in relation to epithelial cells changed: the nearly contiguous bacteriocyte layer building up the outer layer of the midgut tissue present in stage L1 is broken up (Figure 2A). Thus, a characteristic feature of this stage is the presence of scattered bacteriocyte islands in the outer layer of the midgut tissue and a large number of bacteriocytes intercalated between bacteria-free midgut cells. As in the case of L1 larvae no bacteriocytes were found in the epithelial layer lining the gut lumen (Figure 2D-E). This distruption of the layer of bacteriocytes may be due to a strong increase in the size of the gut due to a proliferation of the epithelial cells lining the gut lumen. The same island-like distribution of bacteriocytes has been observed previously in L2 larvae by *in situ *hybridization [4] and could also be seen after staining of actin fibres, which are part of the muscle network surrounding the gut tissue. In these preparations stained clusters of bacteriocytes were visible directly underneath the muscle network enclosing the midgut (Figure 3).

### Bacteriocyte dynamics during metamorphosis

**Figure 4:**
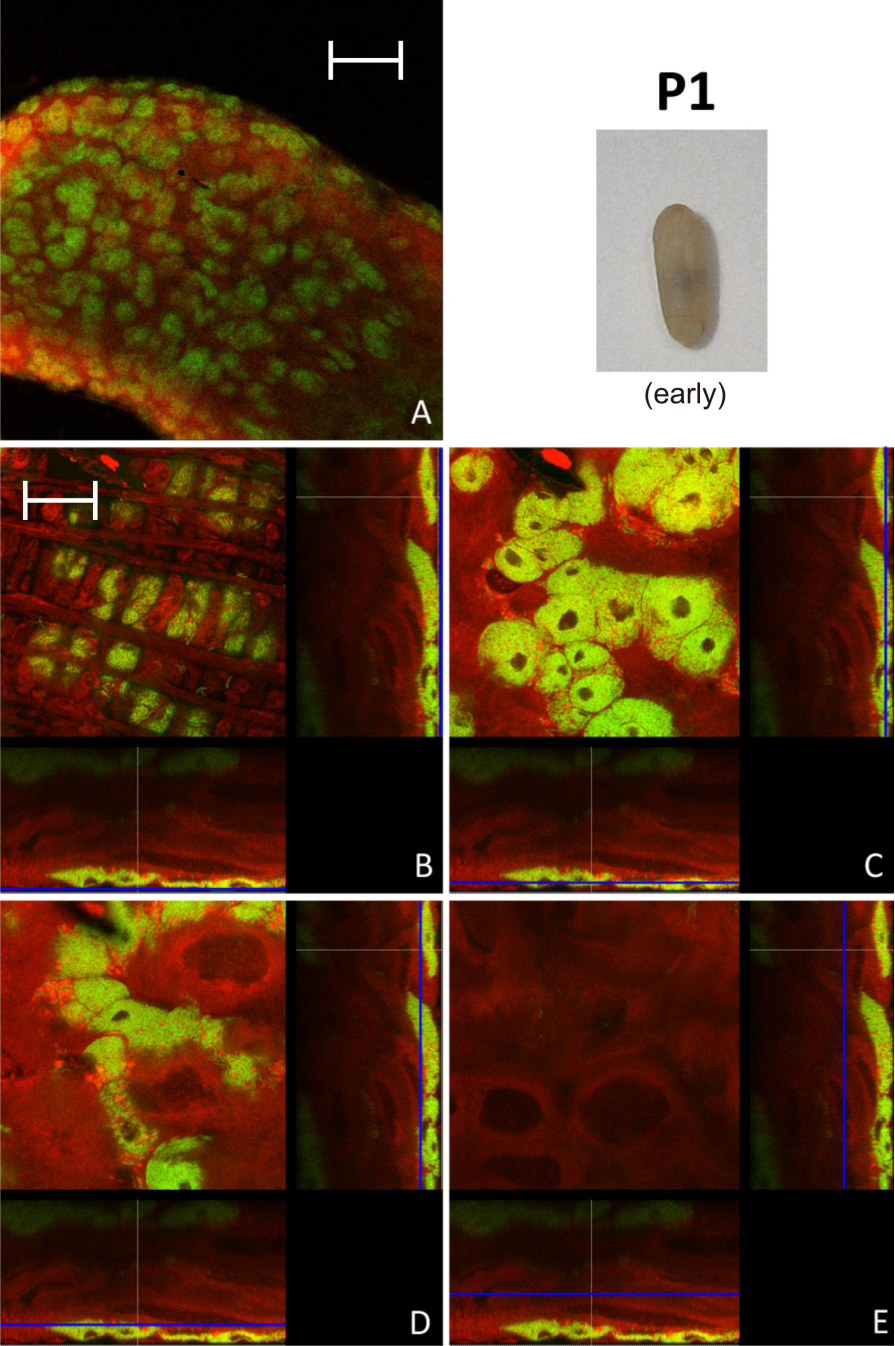
**Early stage P1 pupa**. Overview (A) and detailed images of different optical sections (B - E) of the midgut of a *C. floridanus *pupa (P1) prior to excretion of the meconium by confocal laser scanning microscopy (for further information regarding the composition of the figure see legend of Fig. 1). The distribution of bacteriocytes resembles that of L2 larvae shown in Fig. 2. Green label: The *Blochmannia *specific probe Bfl172-FITC; red label: SYTO Orange 83. The scale bars correspond to 220 μM (A) and 35 μM (B - E), respectively.

**Figure 5:**
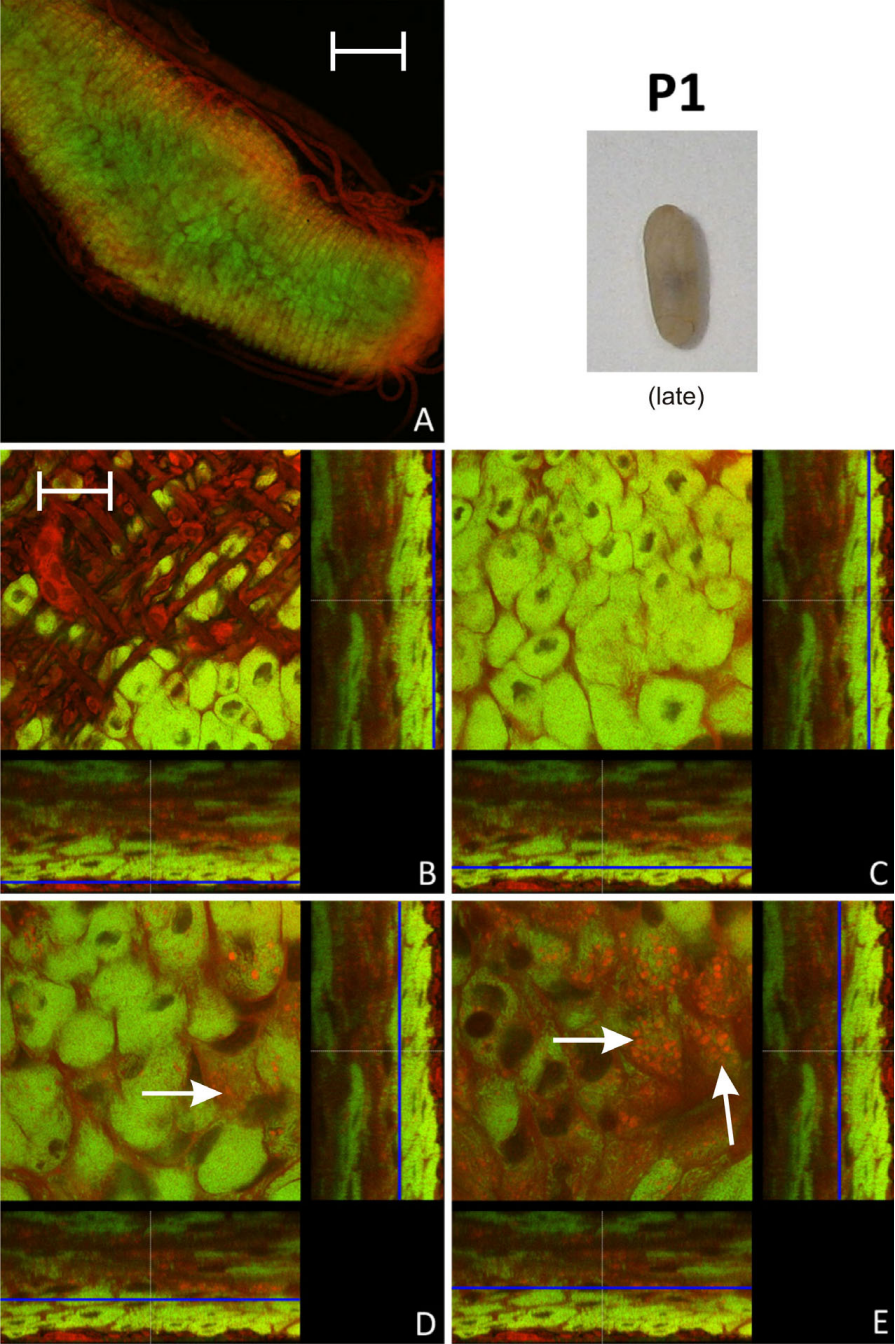
**Late stage P1 pupa**. Overview (A) and detailed images of different optical sections (B - E) of the midgut of a *C. floridanus *pupa (P1) at a later stage than the pupa shown in Fig. 4 by confocal laser scanning microscopy (for further information regarding the composition of the figure see legend of Fig. 1). The bacteriocyte layer encloses the entire midgut (C) and the infection of midgut cells other than bacteriocytes (i.e. cells with large and nucleoli-rich nuclei) is increasingly observed (white arrows in D, E). Bacteria-harboring cells are now found in the epithelial layer bordering the gut lumen (E). Green label: The *Blochmannia *specific probe Bfl172-FITC; red label: SYTO Orange 83. The scale bars correspond to 220 μM (A) and 35 μM (B - E), respectively.

**Figure 6:**
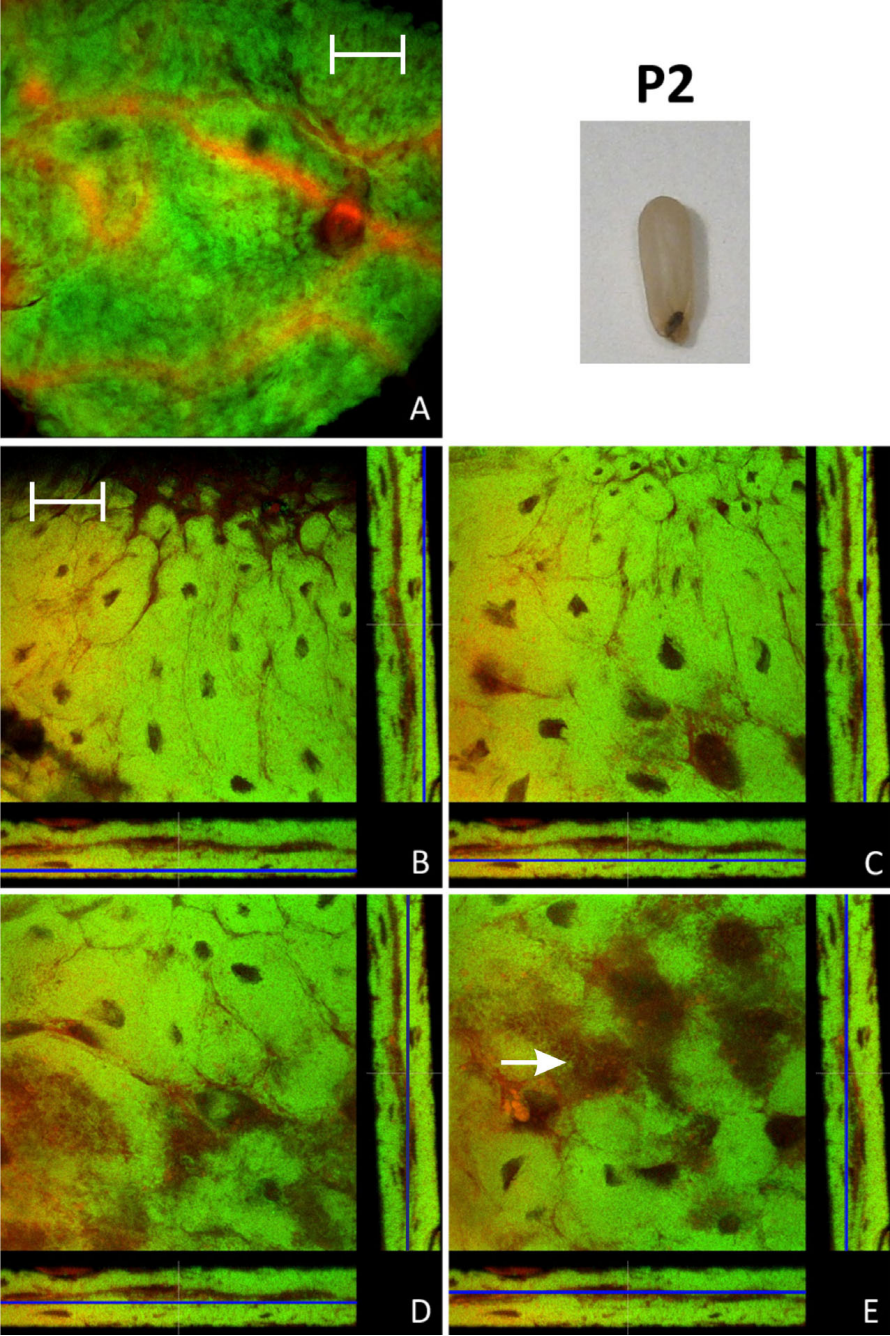
**Pupa of stage P2**. Overview (A) and detailed images of different optical sections (B - E) of the midgut of a pupa after excretion of the meconium (P2) by confocal laser scanning microscopy (for further information regarding the composition of the figure see legend of Fig. 1). Virtually all cells of the midgut harbor *Blochmannia *and the bacteria once more are present in cells with large and nucleoli-rich nuclei (e.g. white arrow in figure part E). Green label: The *Blochmannia *specific probe Bfl172-FITC; red label: SYTO Orange 83. The scale bars correspond to 220 μM (A) and 35 μM (B - E), respectively.

**Figure 7:**
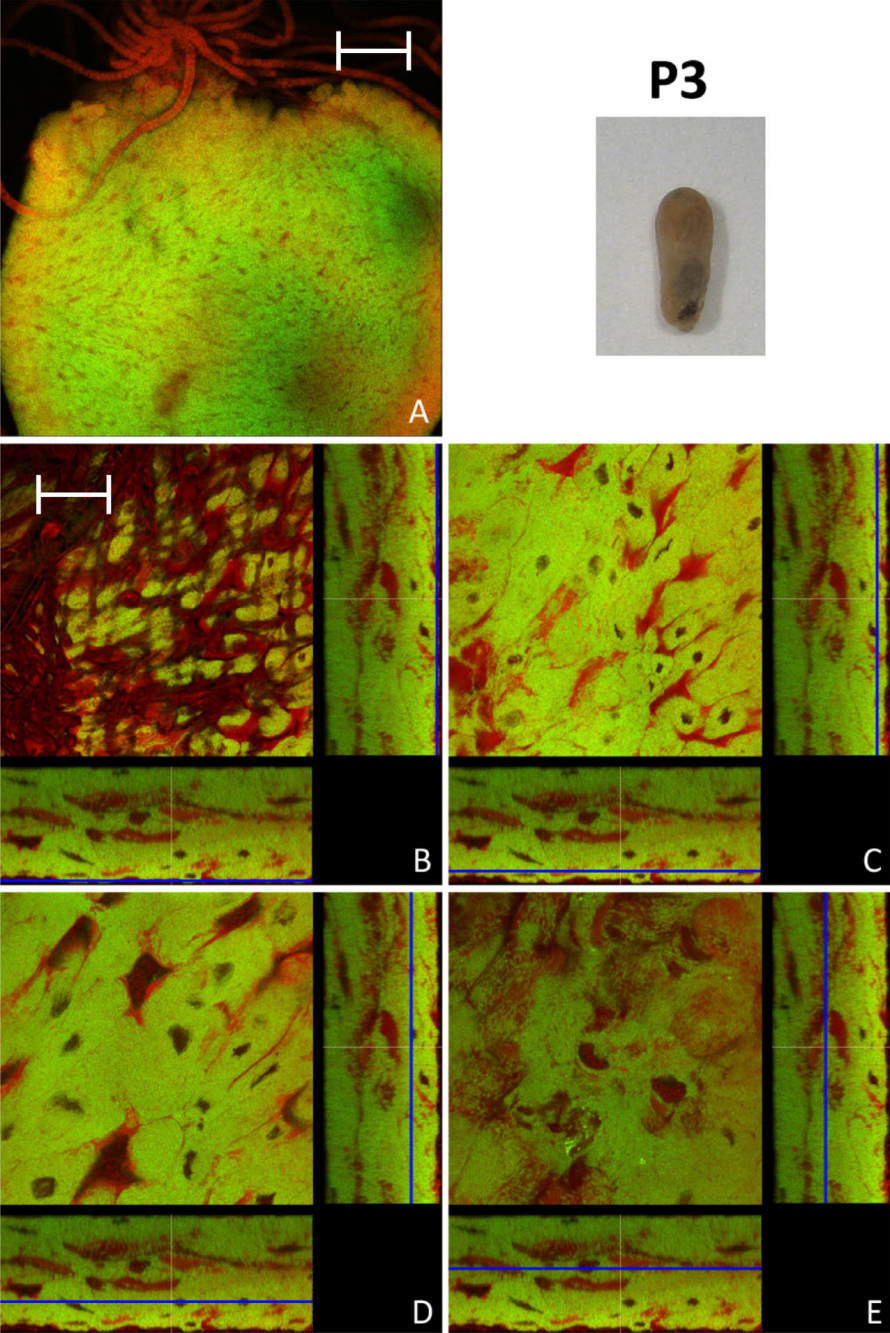
**Pupa of stage P3**. Overview (A, red stained Malpighian tubules are visible on the top of the midgut) and detailed images of different optical sections (B - E) of the midgut of a pupa immediately before eclosion (P3) by confocal laser scanning microscopy (for further information regarding the composition of the figure see legend of Fig. 1). In comparison to P2 (see Fig. 6), a slight increase in bacteria-free midgut cells with large nuclei can be observed. Green label: The *Blochmannia *specific probe Bfl172-FITC; red label: SYTO Orange 83. The scale bars correspond to 220 μM (A) and 35 μM (B - E), respectively.

In early pupal stage 1 prior to the shedding of the remnants of larval midgut tissue and meconium formation, the distribution of bacteriocytes was still island-like as observed in L2 larvae (Figure 4). This is in accordance with recent results, showing that the number of bacteria is relatively stable between these two developmental stages [15]. However, in the late P1 stage there was a massive increase in the number of bacteriocytes relative to epithelial cells resulting again in a nearly contiguous layer of these cells enclosing the epithelial cells lining the midgut lumen (Figure 5). In P1 pupae we also observed cells harboring bacteria that do not resemble typical bacteriocytes due to the larger size of their nuclei and the frequent presence of SYTO-stained vesicles (Figure 5D, E), possibly suggesting bacterial invasion in otherwise bacteria-free enterocytes (see below). The pupal stage 2 is characterized by the shedding of the remnants of larval gut tissue and excretion of the meconium and, consequently, by an alteration of the structure of the midgut (Figure 6). Astonishingly, at this stage virtually all cells were harboring bacteria. Symbionts appeared to be present mainly in bacteriocytes, but, once more, some enterocytes with large nuclei appeared to harbor *Blochmannia *(Figure 6E). Thus, in contrast to larval stages, virtually all cells of the layer lining the gut lumen contained bacteria. With nearly the whole tissue being formed by bacteria-harboring cells the entire midgut can be viewed as symbiotic organ resembling the organ-like bacteriomes of aphids and tsetse flies. This was paralleled by an increase in the absolute number of *Blochmannia *harbored per host [15]. In pupal stage 3 shortly before eclosion bacteriocytes still were the dominating cell type of the midgut, but within the dense bacteria-harbouring midgut tissue again some bacteria-free cells started to appear (Figure 7).

### Bacteriocyte distribution in adult animals

**Figure 8:**
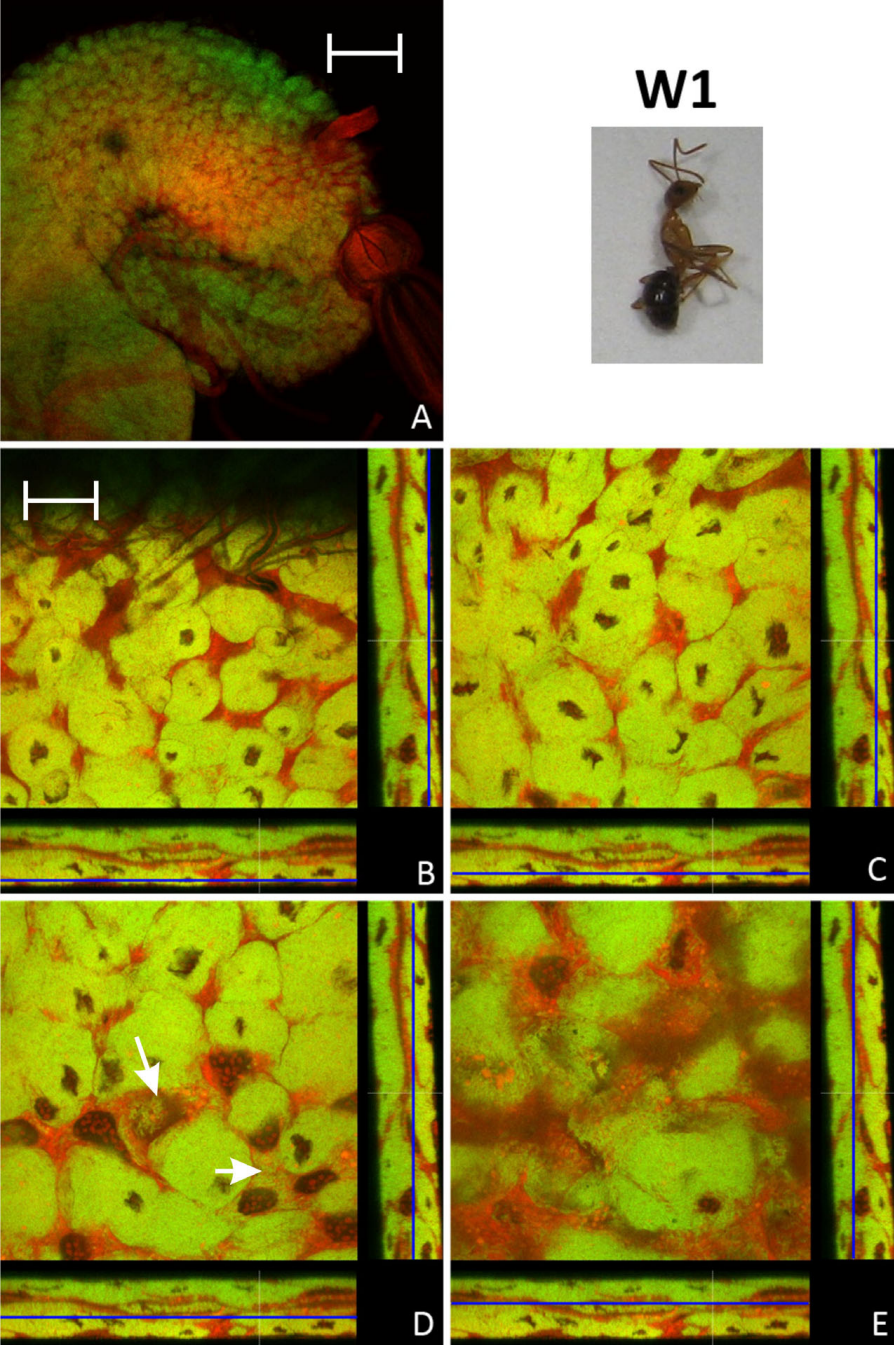
**Imago of stage W1**. Overview (A) and detailed images of different optical sections (B - E) of the midgut of a young worker shortly after eclosion (W1) by confocal laser scanning microscopy (for further information regarding the composition of the figure see legend of Fig. 1). In the overview (A) the proventriculus can be seen on the right side of the midgut. The number of not-infected cells with larger nuclei is increased in comparison to the late pupae stages (Fig. 7). Still there are bacteria in cells which do not resemble typical bacteriocytes (e.g. white arrows in figure part D). Green label: The *Blochmannia *specific probe Bfl172-FITC; red label: SYTO Orange 83. The scale bars correspond to 220 μM (A) and 35 μM (B - E), respectively.

**Figure 9:**
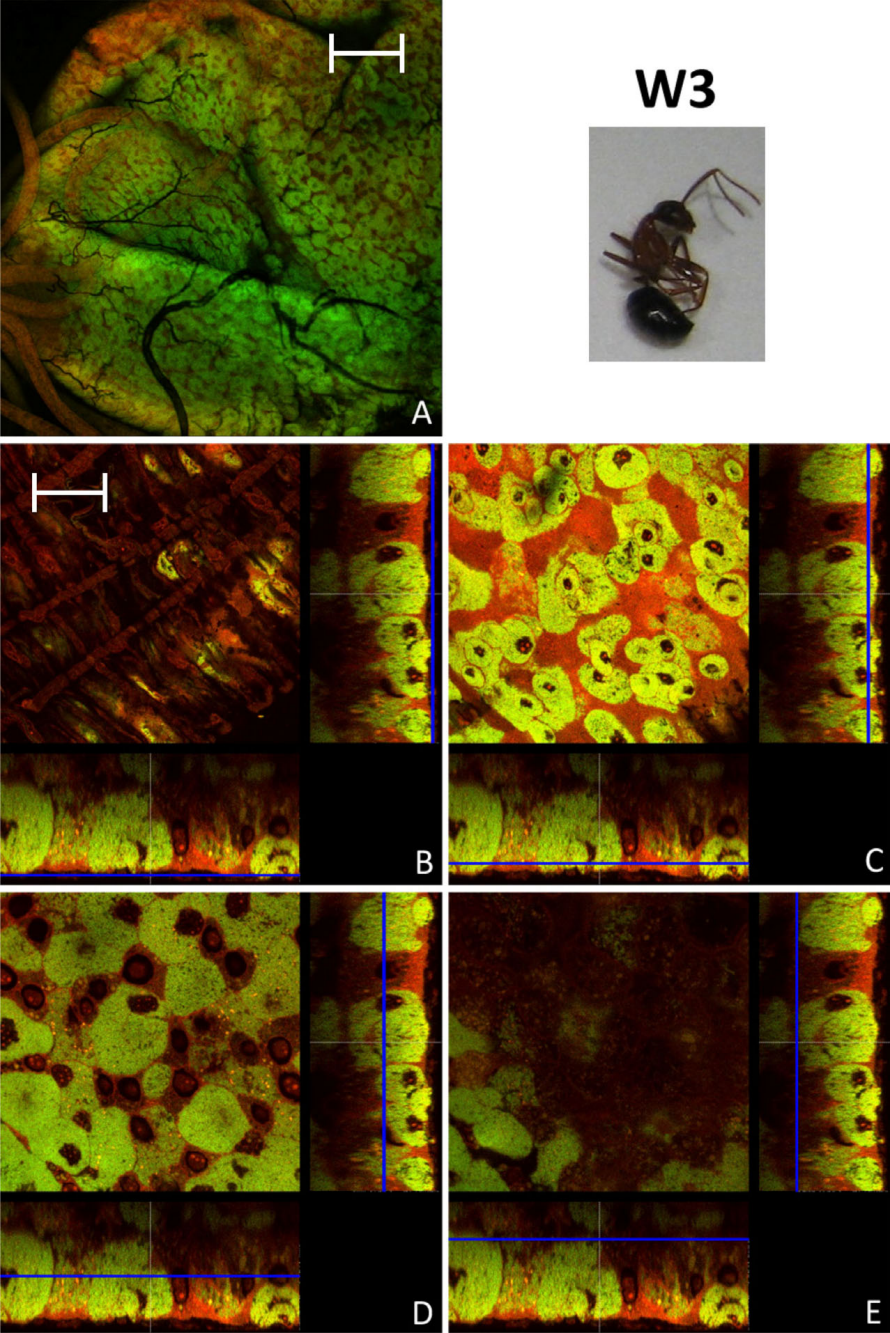
**Imago of stage W3**. Overview (A) and detailed images of different optical sections (B - E) of the midgut of a worker several months of age (W3) by confocal laser scanning microscopy (for further information regarding the composition of the figure see legend of Fig. 1). The proportion of bacteria-free cells is strongly increased, but still there are many bacteriocytes present. Green label: The *Blochmannia *specific probe Bfl172-FITC; red label: SYTO Orange 83. The scale bars correspond to 220 μM (A) and 35 μM (B - E), respectively.

**Figure 10:**
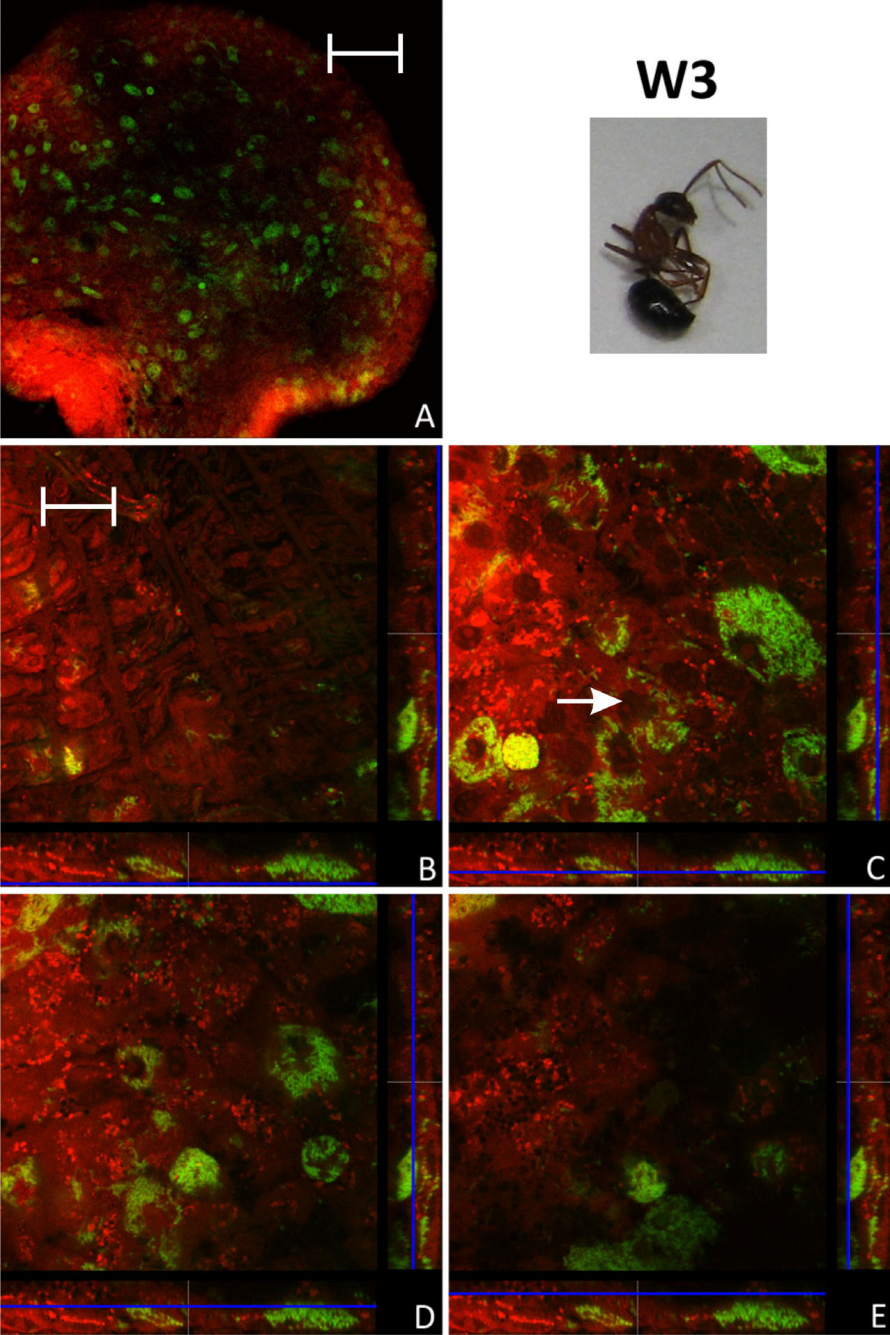
**Imago of stage W3**. Overview (A) and detailed images of different optical sections (B - E) of the midgut of another worker several months of age (W3) by confocal laser scanning microscopy (for further information regarding the composition of the figure see legend of Fig. 1). The number of bacteriocytes is strongly reduced as compared to the worker (W3) shown in Fig. 9. Bacteria present in other cell types than bacteriocytes can be observed (e.g. white arrow in figure part C). Green label: The *Blochmannia *specific probe Bfl172-FITC; red label: SYTO Orange 83. The scale bars correspond to 220 μM (A) and 35 μM (B - E), respectively.

**Figure 11:**
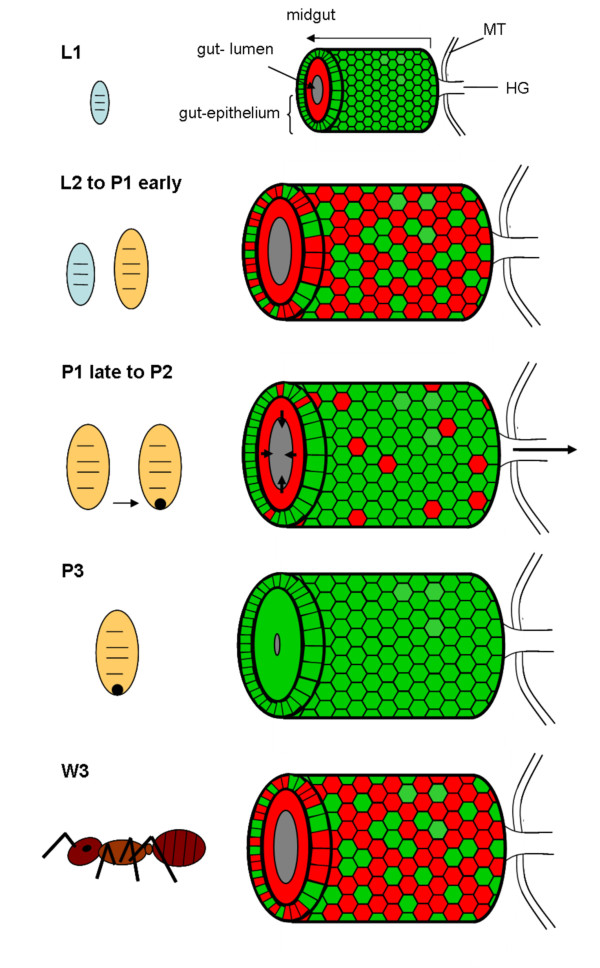
**Schematic overview of distribution of *Blochmannia *in the migut epithelium during host ontogeny, summarizing results of Fig. 1 to Fig. 10**. Red coloured cells are free of *Blochmannia *and green coloured cells are filled with endosymbionts. In small larvae (L1) all cells of the outer layer of the midgut tissue are filled with bacteria, whereas inner layers are devoid of *Blochmannia*. In larger larvae (L2) and pupae directly after pupation (P1 early) the midgut-epithelium strongly expands paralleling the growth of the individual. A large number of cells in the outer cell layer do not contain *Blochmannia *at this stage. During metamorphosis the larval gut epithelium is shed (P1 late to P2) and excreted, forming the meconium (dark spot) in the distal end of the pupal case. During this stage an increased number of cells in the outer layer of the midgut-epithelium harbour *Blochmannia*. In pupae directly before eclosion (P3) the circumference of the gut lumen is very tiny as it is empty. At this stage the whole midgut can be viewed as a bacteriome, since almost all cells forming the midgut-epithelium harbour *Blochmannia*. After eclosion of workers the symbiosis degrades. In old workers (W3) the majority of cells in the outer layer of the epithelium do not contain *Blochmannia *any longer and the inner layer even less so. The circumference of the gut lumen is larger again. MT: Malphigian tubules, HG: hingut.

Young imagines directly after eclosion showed a very similar midgut structure as P3 pupae, although the proportion of bacteria-free cells with large nuclei was increasing (Figure 8). Previously, it was reported that with increasing age the symbiosis appears to degenerate and the number of symbionts strongly decreases. This decrease in symbiont and bacteriocyte numbers was shown for *C. floridanus *queens and workers, but also for workers of *C. sericeiventris *[4,15,16]. The confocal analysis carried out in this study confirmed these findings. However, the situation in workers older than 6 months is quite heterogeneous with regard to bacteriocyte distribution among individuals. In general, as expected, the ratio of bacteriocytes decreases and the midgut structure resembled that of larvae with bacteriocytes being intercalated between midgut cells close to the basal membrane. However, in some of the animals there were still plenty of bacteriocytes present, while in others the symbiosis degenerated dramatically and only very few bacteriocytes dispersed in the midgut tissue could be observed (Figure 9, 10). An illustration of the results described above is presented in Figure 11 which shows schematic drawings of the different developmental stages and the distribution of bacteriocytes therein.

Males are an evolutionary dead end for the bacteria since they cannot be transmitted to the progeny via the spermatocytes [4]. Nonetheless, just as the females, the males may require the endosymbionts for proper development during early life stages. We observed that the distribution of bacteriocytes during developmental stages of males (derived from unfertilized worker eggs) was very similar to that of workers including the fact that the midgut of late pupae was nearly entirely composed of bacteria-harboring cells (data not shown).

**Figure 12:**
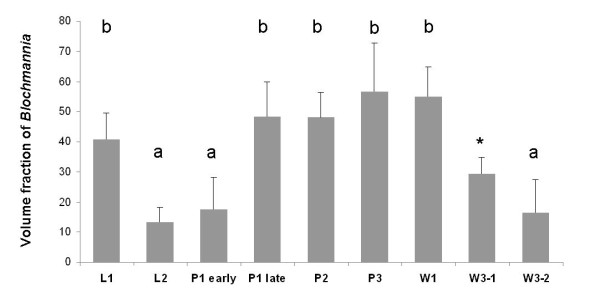
**The figure shows volume fractions of *Blochmannia *symbionts in the midgut tissue of the various developmental stages shown in Fig. 1 to Fig. 10 calculated from the confocal image stacks as described in the Methods section in arbitrary units**. The results show the strong relative decrease of *Blochmannia*-bearing midgut cells between L1 and L2, the strong increase in bacteria-infected cells during the P1 stage and the decrease of bacteria-infected cells in adult animals. Standard deviations are shown as vertical bars on top of the columns. Groups differing significantly at the p < 0.05 level in a Tukey HSD post hoc test are marked with different letters above bars. * W3-1 was not included in the statistical analysis due to small sample size.

Changes in the relative bacterial population density in the midgut tissue of different developmental stages were quantified as described in the Methods section (Figure 12). Volume fractions differed significantly among groups (ANOVA: p < 0.001, F = 13.08, df = 7). The results are in perfect agreement with the optical evaluation described above showing a high proportion of bacteriocytes in L1 (40.84 ± 8.75), when a contiguous bacteriocyte layer is surrounding the midgut (Figure 1). Volume fractions were significantly reduced in comparison to all other developmental stages both in L2 (13.25 ± 4.78) and early P1 pupae (17.63 ± 10.66) when the bacteriocytes have an island-like distribution in the outer layer of the midgut directly underneath of the muscle network due to growth of the midgut tissue in this stage (Figure 2, Figure 3). Also, in older animals the number of bacteriocytes is strongly decreased (29.41 ± 5.51 and 16.44 ± 10.83 for W3-1 and W3-2, respectively; due to small sample size, W3-1 was excluded from ANOVA). The fraction of *Blochmannia*-infected midgut tissue is significantly increased in developmental stages around metamorphosis from late P1 pupae (and 48.34 ± 11.38) to young workers directly after eclosion (W1: 55.04 ± 9.58) (Figure 12).

### Presence of *Blochmannia *in midgut cells other than bacteriocytes

As stated above, some *Blochmannia *may also be found in cells other than bacteriocytes, although the number of bacteria inside these cells appeared to be much lower than in regular bacteriocytes (Figure 5D,E, Figure 6C). The appearance of bacteria-bearing cells not resembling typical bacteriocytes due to their large nuclei was most prominent in pupae around metamorphosis, but occasionally they could also be seen in other developmental stages (Figure 5DE, Figure 10C). An interesting characteristic of such cells was that, frequently, they harbored a much large number of SYTO-stained vesicles than bacteriocytes (Figure 5E). Thus, *Blochmannia *may have the capacity to actively invade into other cell types within the midgut tissue. In agreement with these findings, *Blochmannia *was detected occasionally in midgut cells not resembling bacteriocytes in males of *C. floridanus *and *C. herculeanus *in a previous study [4]. In the cockroach *Blattella germanica *its primary endosymbiont (belonging to the *Bacteroidetes*) is harbored in bacteriocytes lining the fat body. In *B. germanica *it was observed that in nymphal instars the increase in the number of bacteriocytes was not sufficient to explain the strong increase in the number of cells containing endosymbionts. Thus, it was suggested that in these stages bacteria may have invaded fat body cells other than bacteriocytes [28].

Future work must elucidate the nature of these vesicle-containing cells and whether the vesicles may be directly related to the presence of the endosymbionts. It is possible that the observed vesicles are derived from the lysosomal system and may indicate digestion of some bacteria during metamorphosis and during adult stages when the symbiosis degenerates. A similar phenomenon was recently reported for aphids harboring the endosymbiont *Buchnera *within their characteristic symbiosomal vacuoles in bacteriocytes. In these animals about two weeks after ecdysis the bacterial load decreases strongly. A cytochemical analysis revealed the presence of lysosome-like acidic organelles in the bacteriocytes and an upregulation of lysosome-related genes around final ecdysis [22,23]. Electron microscopic analysis of the aphid tissue in these stages revealed a different morphology of the symbiosomes, suggesting degradation of the endosymbionts by the lysosomal system. Digestion of endosymbionts in older ant workers may be reasonable, since the symbiosis does not appear to be of much role in these animals anymore. In fact, in a previous study in *C. sericeiventris *workers *Blochmannia *was occasionally found within vacuoles of host cells [16]. Autophagocytic processes may also be involved in the control of the endosymbiont number keeping it in balance with the host's needs and developmental stages [29].

### Effect of antibiotics treatment on the midgut

**Figure 13:**
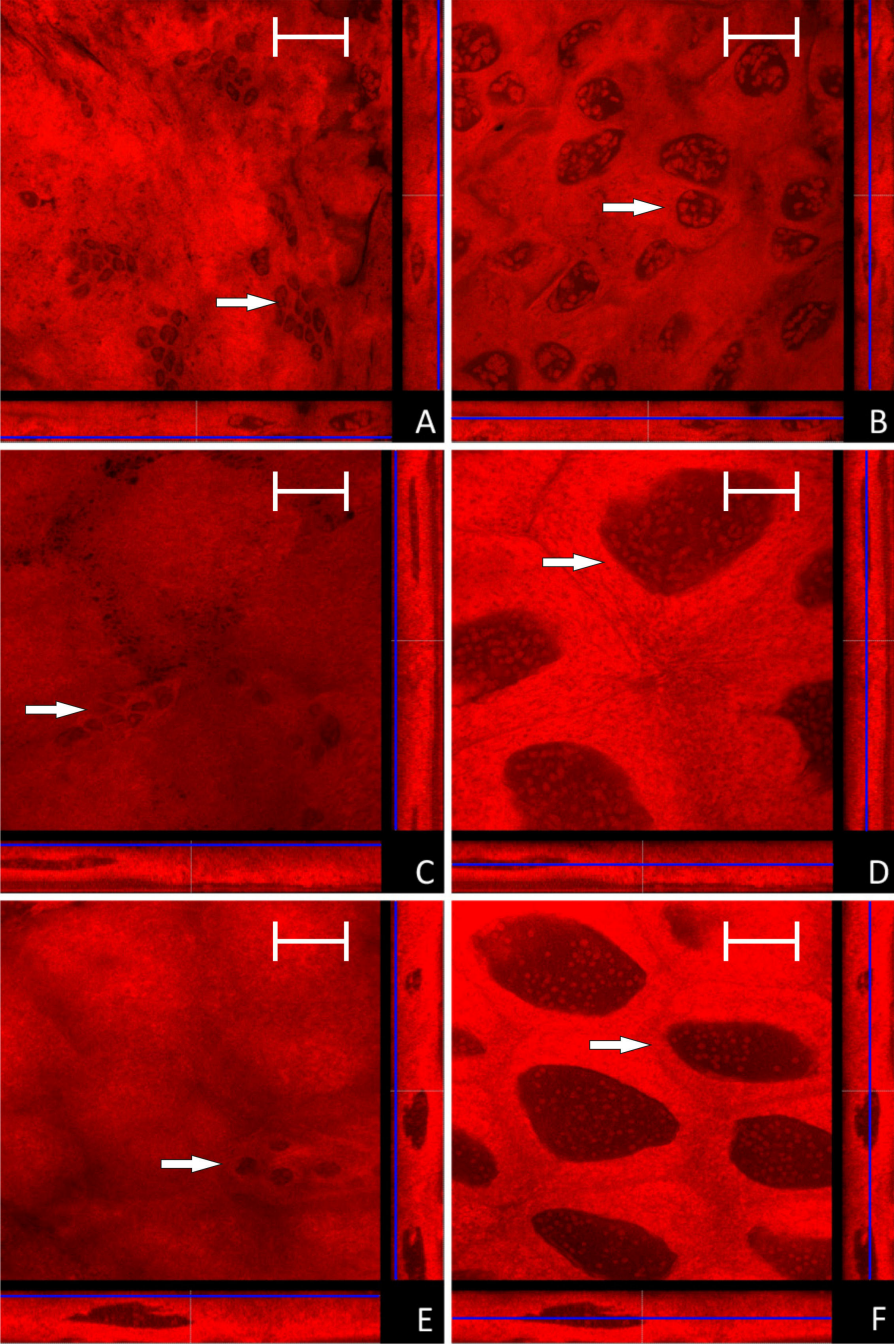
**Confocal micrographs of the midgut of larvae and pupae derived from antibiotics treated queens (for further information regarding the composition of the figure see legend of Fig. 1)**. No *Blochmannia *specific signal is detectable in any of the preparations. Cells resembling empty bacteriocytes are located as small cell clusters on the outer face of the midgut (marked with a white arrow in figure's parts A, C, E), while typical epithelial cells show the characteristic large nuclei (marked with a white arrow in figure's parts B, D, F). A and B: young larvae (L1); C and D: older larvae (L2); E and F: young pupae (P1). Green label: The *Blochmannia *specific probe Bfl172-FITC; red label: SYTO Orange 83. The scale bar corresponds to 35 μM.

Aposymbiotic animals can be obtained by feeding antibiotics to workers or queens. The treatment of queens should reduce the number of endosymbionts transmitted to the next generation via the egg, whereas workers transfer antibiotics directly to the developing larvae via trophallaxis. The breeding success in a colony of an aposymbiotic queen is strongly reduced, but a diet containing all nutrients needed by the brood can counteract the deleterious effect of symbiont loss to some extent [13,14]. Thus, a limited number of aposymbiotic larvae and pupae can be obtained. In none of the investigated larvae and pupae derived from a rifampicin treated queen symbionts could be detected. Nonetheless, in these animals cells characterized by small nuclei (Ø 5 - 8 μm) were found in small clusters of up to 10 cells in the outer layer of the midgut. Based on their small nuclei these cells likely represent empty bacteriocytes (Figure 13). This suggests that, as already shown for aphids [21], the bacteriocytes are formed as part of the normal developmental program of the ant hosts and their generation does not need any bacterial stimulus. However, further analysis is required to unambiguously identify the nature of these cells.

## Conclusions

In conclusion, the data presented here demonstrate that there is a permanent presence of bacteriocytes during pupal stages ensuring that the intracellular endosymbionts are not lost during the complex process of metamorphosis which involves a reconstruction of the inner organs of the insect including the digestive tract. During all stages *Blochmannia *appears to stay within host cells. Thus the maintenance strategy of *Blochmannia *during metamorphosis appears to be fundamentally different from that described for *Candidatus *Erwinia dacicola which shifts from an intra- to an extracellular lifestyle during metamorphosis of the olive fly [24]. Fascinatingly, the strong increase in number of *Blochmannia *and of bacteria-bearing cells during metamorphosis transforms the entire midgut into a symbiotic organ which thus resembles a bacteriome known from other insects. These data confirm the implications of previous experiments which showed an important function of the bacterial endosymbionts for individual animals in particular during pupal stages where their metabolic abilities such as nitrogen recycling very likely are relevant for successful completion of metamorphosis [10,15]. The fact that aposymbiotic larvae have a strongly reduced capacity to complete metamorphosis further underlines this assumption [13]. The massive presence of the symbionts in young workers, whose task is to care for the brood, is in agreement with previous studies which suggested that the endosymbionts may not only contribute to the high individual needs of these animals but may also play a role in upgrading the nutriment provided to the brood by the young workers [13,14]. In the future, it will be important to investigate in detail whether *Blochmannia *indeed has the capacity to invade epithelial cells, which factors are involved in invasion and whether the lysosomal system may play a role in the control of the intracellular bacteria.

## Methods

### Ant culture and stage definition

*Camponotus floridanus *colonies were kept at 25°C with a 12 hour light-dark cycle in artificial nests. The animals were fed twice a week with cockroach pieces (*Nauphoeta cinerea*), Bhatkar agar [30] and honey water (50% w/w) *ad libitum*. The colonies used consisted of at least 2,000 workers. The various developmental stages were defined as follows. L1: small larvae below 2 mm in size; L2: older larvae, approx. 2 - 4 mm in size; P1: pupae before metamorphosis, enclosed in a pupal cocoon but still larva-like in shape; P2: pupae after metamorphosis, with a worker-like shape but still uncolored; P3: pupae with compound eyes visible as dark spots through the cocoon and cuticle of abdomen already partially melanized, shortly before eclosion; W1: callow workers shortly after eclosion which are not yet completely melanized and show no aggressive behavior; W3: adult workers isolated in queen-less worker-groups for at least 6 months. For confocal analysis five animals of each developmental stage were investigated.

### Confocal laser scanning microscopy (CLSM)

Midguts were dissected from individuals and gut content washed out in sterile PBS. Subsequently the midgut samples were fixed on microscopic slides and permeabilized as described previously [13]. Hybridization was carried out by default with FITC-labeled oligonucleotide Bfl172 specific for *B. floridanus *16S rRNA which had been used successfully in a previous study for fluorescent *in situ *hybridization studies [13]. The probe was labeled with the dye at the 5'end as described by the manufacturer (Metabion International AG, Planegg-Martinsried, Germany). Alternatively, red fluorescent Cy3-labeled Bfl172 was used. For CLSM with a Leica DMR laser scanning microscope (Leica Microsystems GmbH, Wetzlar, Germany) these labeled oligonucleotides were applied in combination with SYTO Orange 83 (Molecular Probes Inc.) with a concentration of 2.5 - 5 μM in TE buffer, pH 7.4, resulting in unspecific nucleic acid counterstaining of cytoplasm as well as mitochondria and nuclei after 30 minutes post-FISH incubation and 5 minute washing in TE buffer at room temperature.

For actin-staining 0.5 ng/μl FITC-Phalloidin (Invitrogen Inc.) was used (the *B. floridanus *specific probe was coupled to Cy3 instead of FITC in the respective experiments). The dyes were used according to the manufacturers' protocols. Confocal images were analyzed with the Leica Application Suite Advanced Fluorescence Software (Leica Microsystems GmbH, Wetzlar, Germany). Each of the images shown is representative for a series of preparations from the respective host stage with very similar appearances. For the quantification of *Blochmannia *population densities of ant guts in different larval developmental stages, exemplarily shown in Figure 1 to 10, were calculated as follows: optical sections of gut preparations were recorded by CLSM (see above). Images in the Leica-specific lif file format were opened as ImageJ hyperstacks [31] making use of the LOCI bio-formats plugin (http://loci.wisc.edu/software/bio-formats). The stack corresponding to the FITC channel was thresholded and binarized. The area fraction of labeled *Blochmannia *symbionts was thus measured within each confocal slice. Area fractions were collected for each slice of a stack, summed up, and normalized for the number of slices. The resulting value was termed volume fraction of symbionts (Figure 12). Differences in volume fractions among developmental stages were compared using a one-factorial ANOVA, after homogeneity of variances had been confirmed by Levene's test implemented in SPSS 15.0 (SPSS Inc. Chicago, Illinois, USA).

## Authors' contributions

Conceived and designed the experiments: RG, SS and HF. Performed the experiments: SS. Analysed the confocal images: MJF. Wrote the paper: RG and HF. All authors read and approved the final manuscript.
